# Pharmacotherapy in Patients with Alzheimer-type Dementia Presenting with Behavioral and Psychological Symptoms of Dementia: A Retrospective Chart Review of 102 Patients Available for 12-month Follow-up after Initiation of Treatment

**DOI:** 10.14789/jmj.JMJ22-0024-OA

**Published:** 2023-09-29

**Authors:** MASAYA FUJITA, TAKUYA ISHIZUKA

**Affiliations:** 1 Department of Psychiatry, Hasegawa Hospital, Tokyo, Japan; 1 Department of Psychiatry, Hasegawa Hospital, Tokyo, Japan

**Keywords:** Acetylcholinesterase inhibitor, atypical antipsychotic agents, Alzheimer-type dementia, behavioral and psychological symptoms of dementia, pharmacotherapy

## Abstract

**Objectives:**

Alongside non-pharmacological intervention, pharmacotherapy particularly with atypical antipsychotics is assumed to be effective for behavioral and psychological symptoms of dementia (BPSD).

**Methods:**

This retrospective study investigated the effectiveness and safety of pharmacotherapy including antipsychotics in outpatients or inpatients with BPSD.

**Results:**

Of all Alzheimer-type dementia (AD) patients with BPSD initiating treatment between March and August 2011, a total of 102 patients available for 12-month follow-up comprised the subjects in this chart review. Of these, 68 (66.7%) continued treatment in the ambulatory or inpatient setting, with their MMSE scores improved from 17.3 ± 3.6 at baseline to 18.3 ± 3.53, 17.9 ± 3.80 and 17.0 ± 4.14 after 3, 6 and 12 months, respectively. In contrast, their NPI scores were significantly different from 11.7 ± 11.2 at baseline to 4.86 ± 5.40, 3.56 ± 4.65 and 2.27 ± 3.77 after 3, 6 and 12 months, respectively. Of the 36 inpatients available for follow-up, 27 (75%) on concurrent antipsychotics (chlorpromazine [CP] equivalent, 162.2 mg) at baseline remained on concurrent antipsychotics (CP equivalent, 212.5 mg) after 12 months, while, of the 66 outpatients available for follow-up, 13 (19.7%) on concurrent antipsychotics (CP equivalent, 93.4 mg) at baseline remained on concurrent antipsychotics (CP equivalent, 113.0 mg) after 12 months.

**Conclusions:**

Study results confirmed the effectiveness and safety of the study treatment in Japanese AD patients with BPSD for up to 12 months. How best to incorporate antipsychotics into the treatment of BPSD in clinical settings lies in the hands of us Japanese clinicians.

## Introduction

Now that dementia has emerged as an urgent issue to be addressed with the number of hospitalized patients with dementia was approximately 75,800 in 2020 in Japan^1)^, clinical psychiatrists are often called on to treat not only cognitive dysfunction but also behavioral and psychological symptoms of dementia (BPSD) in patients with Alzheimer-type dementia (AD). BPSD dates back to 1838 when Esquirol defined senile dementia as inclusive of a subtype associated with concomitant emotional disturbance. Only in the 1980s did BPSD become the focus of intensive study. As a consequence, BPSD were assumed to lead to untoward consequences, such as early institutionalization, increased medical costs, decreased quality of life (QOL) of both affected patients and their caregivers, increased stress and excessively impaired performance on the part of their caregivers^2)^. BPSD are reported in many studies to affect a maximum of 97% of dementia patients in nursing homes and communities and to occur with the progression of their dementing illness and highly frequently during a specific period of time^3)^. While, generally, non- pharmacological intervention represents a first treatment of choice for BPSD, BPSD are deemed an indication for pharmacotherapy combined with a non-pharmacological intervention when they are thought likely to adversely affect the QOL of both affected patients and their caregivers or raise safety concerns. However, the US Food and Drug Administration (FDA) issued a warning in April 2005 that elderly dementia patients receiving atypical antipsychotics are at 1.6- to 1.7-fold risk of death compared to those receiving placebo^4)^; as a consequence, the clinical trials of antipsychotics then underway to obtain an indication for BPSD were all discontinued, with the result that, to date, no drugs are available for the treatment of BPSD.

Against this background, therefore, the authors investigated the effectiveness and safety of pharmacotherapy in dementia patients presenting with BPSD in a clinical setting.

## Materials and Methods

This study was deemed exempt from review by the institutional review board of Hasegawa Hospital, Tokyo, Japan as involving only chart reviews and related statistical analyses, with the need to obtain informed consent waived due to the use of anonymized data involving no more than minimal risk to the subjects in this study.

Of all outpatients and the new inpatients with AD treated at Hasegawa Hospital from March and August, 2011, all AD patients newly initiating pharmacotherapy who were available for 12-month follow-up by medical charts were included to retrospectively assess improvements in cognitive symptoms as core symptoms of AD, BPSD, medication adherence, and (reasons for) mediation discontinuation, for 12 months. Outcome measures included: Mini-Mental State Examination (MMSE) for cognitive function; and Neuropsychiatric Inventory (NPI) for BPSD^5)^. On the assumption that the focus should be placed on the assessment of BPSD as incurring a heavier burden on families and caregivers in this study than other outcomes, the study was conducted with NPI as its primary outcome measure, and MMSE, medication adherence and tolerability as its secondary outcome measures. During the study, all patients received galantamine as the only AChEI, but they were also allowed, as the need arose, to concurrently receive any suitable psychotropic agent, except for any other AChEI. A part of patients (18/102, 17.6%) was treated with memantine 10-20 mg/day during the course of the study. At the 12 month, the average dosage of memantine was 18.1 ± 4.0 mg/day (n= 16). The background characteristics of the patients were also explored for their relationship with their course of treatment. All patients had been fully explained about the risks and benefits of the off- label use of psychotropic agents, so that any such psychotropic agent was available for use as needed in patients who gave written informed consent. Patients were judged eligible for study entry if they were diagnosed with probable AD according to the National Institute of Neurological and Communicative Disorders and Stroke and the Alzheimer's Disease and Related Disorders Association (NINCDS-ADRDA) criteria for the clinical diagnosis of AD^6)^ or if they met the operational diagnosis of AD according to the Diagnostic and Statistical Manual of Mental Disorders, Fourth Edition (DSM-IV)^7)^. For statistical analyses, logistic regression model was used. To examine the changes of MMSE and NPI scores between the baseline and each time point, paired t-test was used. In all statistical comparisons, the significance level (two-tailed) was established at *α* = 0.05.

## Results

**Figure 1:**
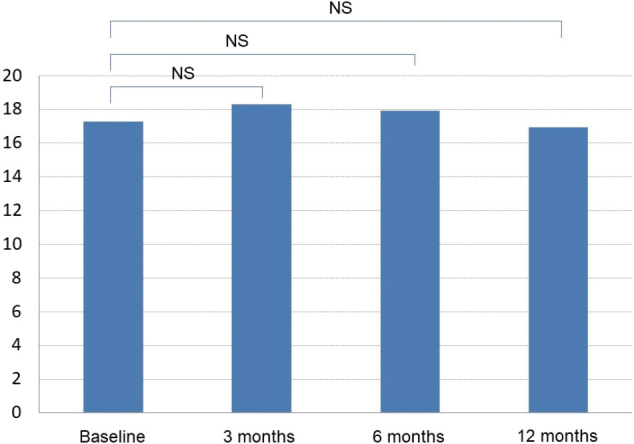
Changes in MMSE scores during follow-up

**Figure 2:**
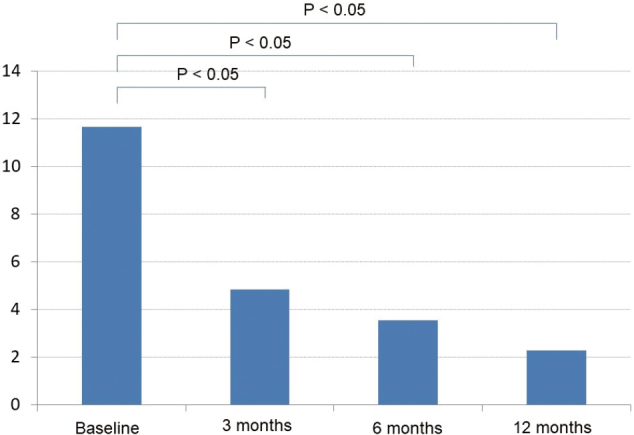
Changes in NPI scores during follow-up

**Figure 3:**
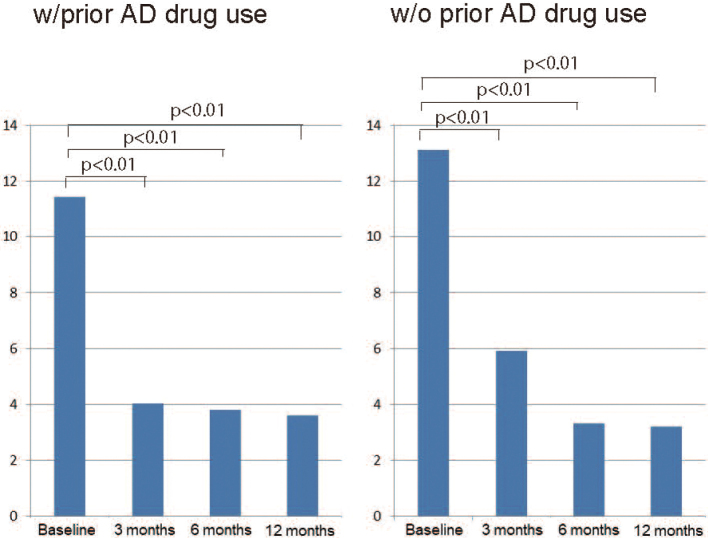
Changes in NPI scores in patients with or without prior AD drug use

**Figure 4:**
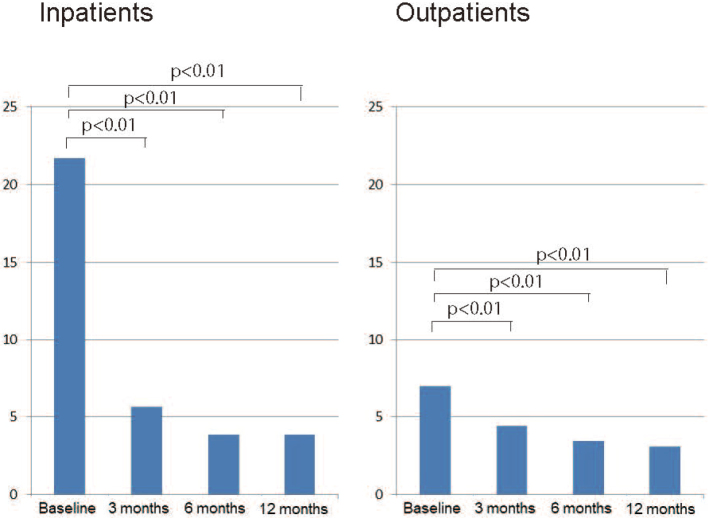
Changes in NPI scores in inpatients and outpatients

Of all AD patients initiating treatment during the period between March and August 2011, a total of 102 patients were available for 12-month follow- up by medical chart review. The subjects consisted of 36 men and 66 women who had a mean age of 77.7 years (men/women, 76.7/78.2 years), a mean (SE) baseline MMSE score of 17.3 (3.6) and a mean baseline NPI score of 11.7 (11.2) (Table 1). Of all subjects, 68 (66.7%) continued with the treatment in the ambulatory or inpatient setting during the 12-month follow-up (Table 2). Their MMSE scores at baseline was 17.3 ± 3.6 and 18.3 ± 3.53, 17.9 ± 3.80 and 17.0 ± 4.14 after 3, 6 and 12 months of treatment, respectively. Their MMSE scores were not significantly different from baseline at any of the time points evaluated (Figure 1). In contrast, their NPI scores were significantly different at 4.86 ± 5.40, 3.56 ± 4.65 and 2.27 ± 3.77 after 3, 6 and 12 months of treatment, respectively from baseline (11.7 ± 11.2) (*P* < 0.05, paired *t*-test) (Figures 2, 3, 4).

**Table 1:** Patient characteristics (n = 102)

No. of men/women	36/66
Mean age	77.7 years (men, 76.7; women, 78.2)
No. of inpatients/outpatients	36/66
No. of patients with/without complications	67/35
No. of patients with/without prior AD drug use	57 (donepezil, 56; rivastigmine, 1)/45
Baseline MMSE score (mean ± SE)	17.29 ± 3.6
Baseline NPI score (mean ± SE)	11.7 ± 11.2

MMSE, Mini-Mental State Examination; NPI, Neuropsychiatric Inventory

**Table 2:** Changes in outcome measures and number of patients on treatment

		Baseline	3 months	6 months	12 months
MMSE	Inpatients	17.94	19.31	19.83	19
	Outpatients	16.97	17.74	17.07	16.118
NPI	Inpatients	17.47	5.64	3.88	3.72
	Outpatients	8.35	4.44	3.44	1.76
No. of patients on treatment	Inpatients	36	18	15	13
	Outpatients	66	75	64	55

MMSE, Mini-Mental State Examination; NPI, Neuropsychiatric Inventory

Of the 36 inpatients available for follow-up, 27 (75%) had been receiving concurrent antipsychotics (chlorpromazine [CP] equivalent, 162.2 mg) at baseline, which included aripiprazole (45%), quetiapine (21%), olanzapine (14%), risperidone extended-release injection (10%), paliperidone (7%) and risperidone (3%), while 8/13 (61.5%) were receiving concurrent antipsychotics (CP equivalent, 212.5 mg) after 12 months of treatment, which included aripiprazole (75%) and paliperidone (25%).

**Figure 5:**
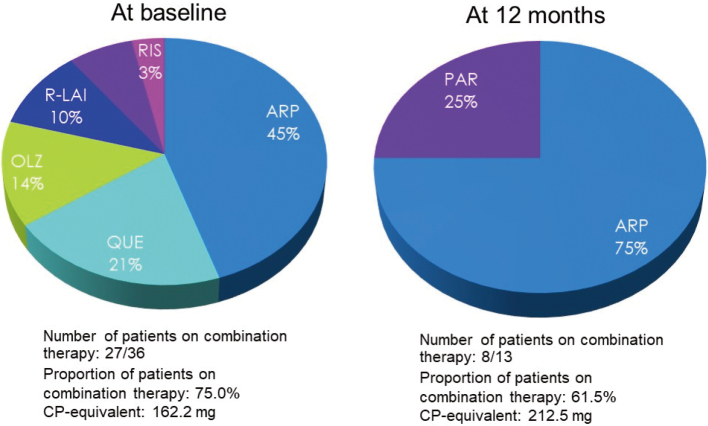
Proportion of inpatients on combination therapy with antipsychotics

**Figure 6:**
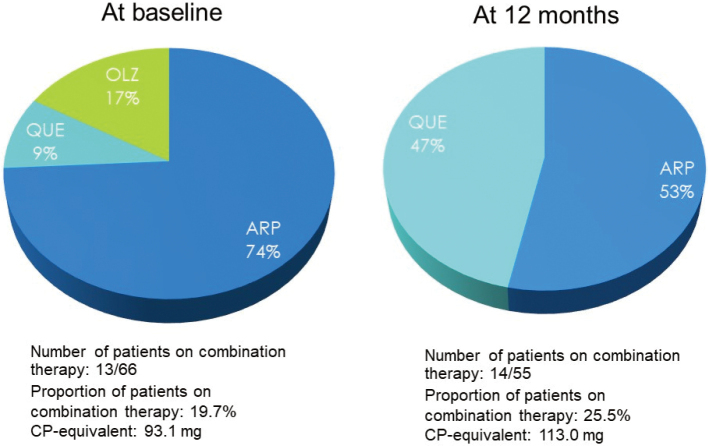
Proportion of outpatients on combination therapy with antipsychotics

In contrast, of the 66 outpatients available for follow-up, 13 (19.7%) had been receiving concurrent antipsychotics (CP equivalent, 93.4 mg) at baseline, which included aripiprazole (74%), quetiapine (9%), and olanzapine (17%), while 14/55 (25.5%) were receiving concurrent antipsychotics (CP equivalent, 113.0 mg) after 12 months of treatment, which included aripiprazole (53%) and quetiapine (47%) (Table 3; Figures 5, 6).

**Table 3:** Inpatients and outpatients on antipsychotics and combination therapy at baseline, 3, 6 and 12 months and transition of patients from inpatient to outpatient antipsychotic treatment

	No. of patients on antipsychotics	Baseline	3 months	6 months	12 months
Inpatients (n = 36)	On an inpatient basis	27	17	12	8
	On an outpatient basis	0	10	6	4
Outpatients (n = 66)	On an inpatient basis	0	0	0	0
	On an outpatient basis	13	13	10	10
Total number of patients on antipsychotics	On an inpatient basis	27	17	12	8
	On an outpatient basis	13	23	16	14
Proportion of patients on combination therapy (%)	On an inpatient basis	75.0	94.4	80.0	61.5
	On an outpatient basis	19.7	30.7	25.0	25.5

## Discussion

In this 12-month follow-up, pharmacotherapy was assessed in a Japanese clinical practice setting for its effectiveness and safety in patients presenting with BPSD, which are particularly associated with AD, of all forms of dementia. Given that the focus of this study was on validating its indications in a broad Japanese AD population in a routine clinical practice setting, but not in a narrow population of patients with AD such as those enrolled in clinical trials, patients were deemed eligible for enrollment if they newly initiated treatment with an AChEI and an antipsychotic agent, and all patients with AD available for 12-month follow-up (n = 102) comprised the subjects in this study.

As regards its effects on cognitive function, anti- AD therapy often results in an initial moderate improvement followed by a gradual decrease thereafter in cognitive function. By the same token, the AD patients in this study showed a moderate improvement in MMSE scores but this improvement was not statistically significantly different from baseline. Again, given that a short-term study such as this should be more focused on the effect of anti-AD therapy on BPSD (primary outcome measure) and its tolerability than on its effects on cognitive function, the subsequent discussion will be focused on its effects on BPSD and its tolerability.

The NPI scores as a measure of BPSD were shown to improve markedly in patients initiating pharmacotherapy in this study. While this may be accounted for in part by the fact that all patients were initially admitted to accommodate their respective condition, here, detailed attention will be devoted to other potential contributing factors.

The improvement in NPI scores varied widely depending on two patient-related factors. First, this had to do with the presence or absence of prior AD drug use. In this study, the improvement in NPI scores was shown to be significant in all patients, irrespective of prior AD drug use, but even more so in those with no prior AD drug use (Figure 3). This was thought likely to be due in large part to the differences in NCI severity at baseline between the patients. Indeed, the NPI scores were shown to be 13.13 at baseline and therefore of much greater severity in those newly initiating the study treatment (i.e., an AChEI plus or minus an antipsychotic agent) compared to 11.45 in those switching to the study treatment from any other AD drug (those with prior AD drug use). This large difference noted at baseline diminished after 3, 6 and 12 months of treatment (5.91/4.03, 3.3/3.8, and 3.2/3.7, respectively, among those with no prior AD drug use/those with prior AD drug use). The greater improvement in NPI scores in the no prior AD drug use group seem to be due to severer disease at baseline. However, despite this large difference in NPI scores at baseline, the MMSE scores at baseline were not significantly different between those newly initiating the study treatment and those switching to the study treatment at 18.55 and 16.32, respectively, suggesting that the severity of BPSD as assessed by NPI scores was not necessarily concurrent or consistent with the decline of cognitive function.

Another background factor thought likely to have affected the changes in NPI scores was the difference in patient status as inpatients and outpatients. As in those newly initiating the study treatment and those switching to the study treatment from previous therapy, the NPI scores differed widely between the inpatients and outpatients, with this difference being even larger than that between those newly initiating the study treatment and those switching to the study treatment from previous therapy. Again, the NPI scores were shown to be 21.72 at baseline and therefore of much greater severity in inpatients than in outpatients (7.00), while this large difference noted at baseline diminished after 3, 6 and 12 months of treatment (5.63/4.43, 3.88/3.44 and 3.86/3.10, respectively, among inpatients/outpatients). This is in complete agreement with the observation in our clinical practice that in most cases, AD patients are newly admitted to our hospital at the request of their caregivers (families) who have had difficulty managing their BPSD. However, despite this large difference in NPI scores at baseline, the MMSE scores at baseline were not significantly different between inpatients and outpatients at 17.93 and 16.97, respectively, thus confirming that the severity of BPSD as assessed by NPI scores was not concurrent or consistent with the decline of cognitive function.

Apart from this, the significant improvement in NPI scores achieved with the study treatment may also be accounted for pharmacologically. The NPI scores improved remarkably in inpatients and those newly initiating pharmacotherapy; due to their greater NPI severity at baseline, however, these patients tended to receive antipsychotics and/or mood stabilizers concurrently with the AChEI (27/36 inpatients and 20/45 patients newly initiating treatment). Furthermore, given that, of the 10 behavioral domains in NPI, “delusions” and “aberrant motor behavior” were significantly improved from baseline in these patients, the marked improvement in NPI scores could be accounted for in large part by the effects of concurrent antipsychotics and/or mood stabilizers among these patients.

In contrast, the NPI scores significantly improved in those switching to the study treatment (i.e., those with prior AD drug use) who had been shown to be of lesser NPI severity at baseline (*P* < 0.05, paired *t*-test) and tended to improve in outpatients newly initiating treatment who had also been shown to be of lesser NPI severity at baseline (mean NPI score, 7.00). Of note, only 20 of the 58 patients switching to the study treatment and 13 of the 66 patients newly initiating treatment had been receiving antipsychotics and/or mood stabilizers, respectively. Thus, it was thought unlikely that the marked improvement in their NPI scores were due to these concurrent medications and quite likely that the improvement in their NPI scores was primarily due to the effect of the AChEI in improving BPSD.

As for evidence for antipsychotic efficacy, the Clinical Antipsychotic Trials of Intervention Effectiveness-Alzheimer's Disease (CATIE-AD) were conducted in 421 AD patients who presented with psychotic symptoms (e.g., delusions and hallucinations), aggression and irritability to evaluate the efficacy of atypical antipsychotics (i.e., olanzapine, quetiapine, risperidone) versus placebo, demonstrating that the atypical antipsychotics were not superior to placebo and offered no benefit over placebo, given their adverse effects^3)^. On the other hand, Yunusa et al. conducted a network meta-analysis in 2019 of clinical trials conducted to date^8)^ to evaluate the relative benefits and safety of atypical antipsychotics in the treatment of BPSD, and demonstrated the efficacy of atypical antipsychotics (aripiprazole, quetiapine, and risperidone) on BPSD. By the same token, this study provided enough evidence for the effectiveness of atypical antipsychotics in AD patients with BPSD, given that the study treatment led to a significant improvement in their NPI scores and significantly improved “delusions” and “aberrant motor behavior”, of the behavioral domains in NPI, among the study subjects.

After the FDA warning^4)^, the J-CATIA study was performed in Japan, in which 10,079 Japanese patients with AD were followed up for 24 weeks^9)^. In this study, 71.4% were taking atypical antipsychotics (quetiapine, risperidone, olanzapine, aripiprazole, and others) and others were taking typical antipsychotics (tiapride, sulpiride, and levomepromazine). Arai et al. reported that patients with AD for whom antipsychotic treatment was started during the follow up period had increased mortality. The daily chlorpromazine equivalent doses were not higher in the patients who died than the whole exposed group^9)^.

Though there are indications that antipsychotic agents are associated with the risk of increasing mortality, they exert certain effects on selected BPSP (e.g., hallucinations, delusions, and agitation) and therefore cannot be dispensed with in real-world clinical settings. Thus, the authors conclude that while antipsychotics may be used, as the need arises, in AD patients whose BPSD are so severe as to require inpatient care, attention should be focused on reducing their dose or discontinuing them as their symptoms become alleviated, as well as on using antipsychotics with full understanding of their characteristics and each patient's symptoms and integrating them nicely with non-pharmacological therapy.

This study has some limitations. First, it included only an insufficient number of AD patients. Second, it was conducted as a retrospective study with no comparative control group. Third, BPSD were not evaluated in those with any other form of dementia than AD. Given the paucity of clinical evidence in Japan for the effectiveness of antipsychotic agents against BPSD, however, this study may represent a valuable addition to the literature, in that it corroborated the earlier findings reported overseas on their efficacy against BPSD.

## Conclusions

The authors followed up the medical charts of AD patients newly initiating pharmacotherapy with the anti-AD agent galantamine and concurrent antipsychotic agents in Japanese AD patients with BPSD, evaluated a total of 102 patients who were available for 12-month follow-up, and confirmed the effectiveness and safety of the study treatment for up to 12 months. While how best to incorporate antipsychotics into the treatment of BPSD in clinical settings lies in the hands of us Japanese clinicians, accumulation of further clinical evidence is eagerly awaited in the years to come.

## Funding

No funding was received for this study.

## Author contributions

MF and TI both contributed equally to the design and conduct of the study. MF performed the statistical analysis of the study data and TI oversaw the statistical analysis and interpretation of the study data. MF wrote and revised the manuscript and TI participated in the writing and revision of the manuscript. The authors have read and approved the manuscript for publication.

## Conflicts of interest statement

The authors declare that they have no known competing financial interests or personal relationships that could have influenced the work reported in this paper.
